# The Genome Sequence of *Avibacterium paragallinarum* Strain CL Has a Large Repertoire of Insertion Sequence Elements

**DOI:** 10.1128/genomeA.00152-17

**Published:** 2017-04-13

**Authors:** Guillermo Horta-Valerdi, Maria Patricia Sanchez-Alonso, Victor M. Perez-Marquez, Erasmo Negrete-Abascal, Sergio Vaca-Pacheco, Ismael Hernandez-Gonzalez, Zulema Gomez-Lunar, Gabriela Olmedo-Álvarez, Candelario Vázquez-Cruz

**Affiliations:** a Centro de Investigaciones en Ciencias Microbiológicas, Instituto de Ciencias, Benemérita Universidad Autónoma de Puebla, Puebla, Puebla, Mexico; b BIOVETSA Laboratorios, Tehuacán, Puebla, Mexico; c Carrera de Biología, Facultad de Estudios Superiores de Iztacala, UNAM, Tlalnepantla, Edo de México, Mexico; d Centro de Investigación y de Estudios Avanzados del IPN, Campus Irapuato, Irapuato, Guanajuato, Mexico

## Abstract

The draft genome sequence of *Avibacterium paragallinarum* strain CL serovar C is reported here. The genome comprises 154 contigs corresponding to 2.4 Mb with 41% G+C content and many insertion sequence (IS) elements, a characteristic not previously reported in *A. paragallinarum*.

## GENOME ANNOUNCEMENT

Infectious coryza is a globally distributed disease produced by *Avibacterium paragallinarum*, a Gram-negative bacterium that causes serious economic losses in poultry production (1) because this microbe harbors an impressive battery of virulence factors (2, 3, 4). Classical serotyping of *A. paragallinarum* includes A, B, and C serovars (1). A recent emergence of atypical strains of *A. paragallinarum* in outbreaks of infectious coryza has been reported (5). Among the promising strategies for the control of this disease, determination of DNA sequences of the *A. paragallinarum* genome is the most outstanding strategy to study its pathogenesis at a molecular level. To date, only three genome sequences of the strains AVP72, 221, and JF4211 of *A. paragallinarum* have been assembled in scaffolds (6, 7, 8). To investigate the genomic basis of the pathogenesis of this bacterium, the *A. paragallinarum* CL strain serovar C was used for whole-genome sequencing, which revealed an enhanced richness of insertion sequence (IS) elements that could explain the reduced assembly of these genomes.

The genomic DNA of *A. paragallinarum* str. CL was extracted from fresh bacterial cultures and purified using phenol for sequencing using a whole-genome shotgun strategy with Roche GS FLX System 454 Sequencing Technology (Branford, CT, USA) (9). Reads averaging 600 bp in length were obtained. The reads were filtered based on quality and subsequently used to assemble contigs with Mira software (10).

The assay reached 118-fold coverage, which generated 340 contigs. To identify unique genes and obtain short sequences, such as tRNAs, a review with the Rast platform (11) was performed, and 154 scaffolds were obtained. The largest contig was approximately 118,593 bp, and the minimum contig was 1,209 bp. The genome of *A. paragallinarum* strain CL was estimated to be 2,410,835 bp in length with an average G+C content of 41.3%. Automatic gene prediction and annotation were performed using the NCBI Prokaryotic Genome Annotation Pipeline (http://www.ncbi.nlm.nih.gov). In total, the CL genome comprised 2,512 coding sequences, among which 2,267 protein-encoding genes were predicted, with a 96% coding percentage, and 1% of the genes were RNA-coding genes. The average gene length was 959 bp. Rast tools revealed 2,502 genes distributed among 27 subsystems and 576 hypothetical proteins. A total of 142 putative proteins were related to integrases or transposases, and another 117 putative proteins were related to phage genes. The Mu-phage genes were vestigial (12). Moreover, 20 copies of RNA were predicted and distributed among at least 22 loci. We identified seven predicted copies of each of the 5S and 23S rRNA genes, five copies of 16S rRNA genes, and 51 tRNA genes. At least 270 insertion sequences or repeated sequences were identified and grouped into six superfamilies based on similarity.

### Accession number(s).

This whole-genome shotgun project has been deposited at DDBJ/EMBL/GenBank under the accession number LAEN00000000. The version described in this paper is the first version, LAEN01000000.
